# Supplementary material for: The shared experiences of insured members and the uninsured in health care access and utilization under Ghana’s national health insurance scheme: Evidence from the Hohoe Municipality
Source: PLoS One. 2020 Dec 23;15(12):e0244155. doi: 10.1371/journal.pone.0244155 (PMC7757881; doi:10.1371/journal.pone.0244155)
Supplement: S1 File — (DOCX) [file pone.0244155.s001.docx]

# Experiences of National Health Insurance Scheme (NHIS) Subscribers and Non-Subscribers in Accessing and Utilizing Health Care in the Hohoe Municipality

## Appendix D. Data collection tool

**Interview guide**

This research is being conducted by a final year student of the University of Health and Allied Sciences, School of Public Health - Hohoe. The purpose of the study is to examine the experiences of NHIS subscribers and non-subscribers in healthcare access and based on insurance status as well as provider’s perspective about their clients’ experiences.

The results from this study will be used to inform policy decisions on ensuring access and use of health services in the Hohoe municipality. Your participation is highly appreciated and you are assured that any information you provide will be confidentially kept without personally identifying you in any reports. Thank you.

**PART A-Socio-Demographic Characteristics**

1. Name of respondent

2. Age of respondent

3. Gender

4. Occupation of respondent

5. Highest Educational level

6. Residence

7. Marital status

8. Ethnicity

9. Nationality

10. Religion

11. Type of Family (nuclear/extended)

12. Insurance Status

**PART B-Respondents’ Awareness of the NHIS and its Operations**

1. Have you ever heard of the NHIS? (What is it)?

2. What benefits are there for a person who subscribes to the NHIS?

3. Between an NHIS subscriber and a non-subscriber, who do you think will have easier access to health care and why?

**PART C- Exploring Possible Barriers and Disparities in Health Care Accessibility**

**Subscriber**

1. To what extent has the NHIS made a difference in your access to health care and do you think those without the NHIS card have challenges in accessing health care?

2. When was the last time you utilised the health facility and what was your experience?

3. Did you pay any extra money at the health facility? What did you pay for?

13. Will you recommend the NHIS to a friend?

14. How far is the NHIS office located from your home?

15. Do you find the NHIS premium expensive or less expensive?

16. What recommendations do you have to make to better the NHIS?

**Non-subscriber**

1. Why are you not subscribed to the NHIS?

2. How close is the NHIS office to your house?

3. What do you think about the NHIS premiums?

4. What are your experiences in accessing health care and paying out-of-pocket? Would it have been better or worse if you were to be insured?

6. When was the last time you utilised the health facility and what was your experience?

7. How did you pay for the services rendered to you?

13. What do you think are the reasons why some people are not subscribed to the NHIS?

**PART D- Views of Service Providers on the Experiences of NHIS Subscribers and Non-Subscribers in Accessing and Utilising Health Care Services.**

1. What are your experiences of providing health care services under the NHIS and out-of-pocket payments?

2. Has the NHIS made any difference in equitable access to health care in the municipality?

3. How will you describe the experiences of insured and uninsured clients in accessing healthcare?

4. How do clients pay for health services in your facility?

**PART E – Barriers to Health Care Utilisation and Access by both Subscribers and Non-Subscribers**

1. How close is the health facility to you and what challenges do you face in accessing the health facility?

**PART F – Motivating Factors to Health Care Utilisation and Access by both Subscribers and Non-Subscribers**

1. What motivates you to use the health facility?
