# Supplementary material for: The shared experiences of insured members and the uninsured in health care access and utilization under Ghana’s national health insurance scheme: Evidence from the Hohoe Municipality
Source: PLoS One. 2020 Dec 23;15(12):e0244155. doi: 10.1371/journal.pone.0244155 (PMC7757881; doi:10.1371/journal.pone.0244155)
Supplement: Attachment — Submitted filename: Comments.pdf [file pone.0244155.s002.pdf]

General: A good and interesting study

Abstract

Summarised the research aim and objectives with concise method and findings stated.

Introduction

Line 1: change "systems" to "scheme".

The manuscript requires editing to correct some typo error such as in L3 in Introduction "Low-And Middle-Income Countries (LMICs)" should be "and". Line 7 too.

On page 6, kindly remove the statement "*The study sought to explore the experiences of NHIS insured and uninsured in accessing and utilizing health care services*" it is a repetition.

Methods

Adequately described

Kindly describe any saturation experienced during data collection

Result

On page 17, I think it will be better to have reasons for Non-subscription and Recommendations to make the NHIS better to clear sub-heading as it was done in barriers to Health Care Access and Utilization by NHIS Insured and Uninsured and others findings.

Kindly clearly state finding obtained from the health professionals in the result section. This seems to be missing in the report.

Discussion

As in the result section, the sub-sections in this section seems to focus more the insured and uninsured participants, kindly incorporate a bit of health professionals' opinion has this was stated in the methodology.
