# Supplementary material for: The shared experiences of insured members and the uninsured in health care access and utilization under Ghana’s national health insurance scheme: Evidence from the Hohoe Municipality
Source: PLoS One. 2020 Dec 23;15(12):e0244155. doi: 10.1371/journal.pone.0244155 (PMC7757881; doi:10.1371/journal.pone.0244155)
Supplement: Attachment — Submitted filename: Response to Reviewers.docx [file pone.0244155.s003.docx]

Dear Editor-In-Chief,

*Subject:* **Revised submission of manuscript for consideration**

We found the reviewers comments to be very helpful in guiding us through what became an extensive revision process of a ‘minor revision’. While the main objectives of writing the article remain intact, there has been some few movements of paragraphs due to the reviewers’ comments. We also acknowledge that much of the text on health providers’ views is new in order to meet the suggestions of one of the reviewers. We are grateful for reviewing our paper and providing us with the opportunity to resubmit the revised manuscript. Enclosed to this letter is our revised manuscript titled: *“The shared experiences of insured members and the uninsured in health care access and utilization under Ghana's National Health Insurance Scheme: Evidence from the Hohoe Municipality*”.

In revising the manuscript, we took into account all reviewers’ suggestions and constructive criticisms of our initial submission. A point-by-point explanation of how we addressed the comments raised by each reviewer is detailed below. We believe that the reviewers’ comments helped us to make a critical revision of the manuscript, which will be interesting for readers in the wider scientific community.

All authors have read and approved this revised manuscript, and agreed with the publication of their names. There are no conflicts of interest.

We look forward to hearing from you on this matter.

Sincerely,

Adam Fusheini (PhD)

(Corresponding Author)

**Detail Revision notes on revised Manuscript**

## **Manuscript ID**: PONE-D-20-18400

**Title:** The shared experiences of insured members and the uninsured in health care access and utilization under Ghana's National Health Insurance Scheme: Evidence from the Hohoe Municipality

Below, we explain how we have addressed each of the comments of the reviewers.

**Journal Requirements**

1. *Please ensure that your manuscript meets PLOS ONE's style requirements, including those for file naming. The PLOS ONE style templates can be found at*

**Response:** We have reformatted the manuscript in line with PLOS ONE’s style

2. *Please include additional information regarding the survey or interview guide used in the study and ensure that you have provided sufficient details that others could replicate the analyses. For instance, if you developed a questionnaire as part of this study and it is not under a copyright more restrictive than CC-BY, please include a copy, in both the original language and English, as Supporting Information*

**Response:** Additional information is provided on the interview guide used on pages 10-11, lines 241-249 of the revised/tracked changes version. Also, a copy of the interview guide has been included as supporting information.

3. *We note that "Map of Hohoe Municipal" in your submission contain [map/satellite] images which may be copyrighted.*

**Response:** The “map of Hohoe municipal” has been removed from the revised manuscript.

**REVIEWER #1:**

**Comment 1**: *Under the introduction, please note that not all developed countries "have successfully ensured optimum levels of healthcare for their entire populace," as has been stated, an example of this is the United States where a large proportion of the population is still uninsured and so have no usual access to care*.

**Authors’ response:** The sentence has been corrected and “some” has been inserted to indicate that not all developed countries have successfully ensured optimum healthcare for their entire population. The sentence now reads as:

*“While* ***some*** *developed countries have successfully ensured…”* in **line 17 of page 1** of the tracked changes version of the manuscript.

**Comment 2:** "bedevil" not an appropriate term to use in a technical writing as in this manuscript.
**Authors’ response:** “bedevil” has been replaced with “face”.

*“Health care accessibility and utilization remains limited as a result of financial and socio-cultural challenges that* ***face*** *such economies”* in **line 20 on page 1** of the tracked changes version of the manuscript.

**Comment 3:** Why are the % in parenthesis? E.g. about (55%)?
**Authors’ response**: The wrongly placed parentheses have been removed. The text now reads as:

*“About* ***55%*** *of the population are engaged in farming. Twenty-five percent are also involved in trading,* ***15%*** *in livestock rearing and* ***5%*** *engage in other…”* in **lines 203-205 on page 9** of the tracked changes version of the manuscript.

**Comment 4:** (25%) ---Since this figure appears at the beginning of a sentence, the accepted norm is to write it out in words.

**Authors’ response:** The figure has been written in words as follows:

***“Twenty-five percent*** *are also involved in trading, 15% in livestock rearing and 5% engage in other”* in **lines 204-205** of the tracked changes version of the manuscript.

**REVIEWER #3**

**Comment 1:** On page 1, I would recommend including the word 'some' in the second paragraph to state, "While some developed countries have successfully ensured optimum levels of healthcare for their entire populace ..."

**Authors’ response**: The sentence has already been corrected in **line 17 of page 1** of the tracked changes version of the manuscript

**Comment 2:** On page 3, you use the abbreviation GHS but I do not see this abbreviation defined previously in the text. Though this may have been an oversight on my part, I could not find it. You also translate this currency into $ but not define which dollar you are referring to... United States of America (US dollars) or another dollar?

**Authors’ response**: The abbreviation GHS has been defined and the currency conversion to dollar has been specified as United States of America dollar (US dollar) as follows:

*“The annual premium varies between twenty-five Ghana Cedis (GHS25) (approximately US$4.30) and GHS46 (US$7.91) between the low- and upper- income groups. Those who contribute to SSNIT only need to pay a registration fee of about GHS5 (US$0.86).”* in **lines 42-44 on page 2** of the tracked changes version of the manuscript

**Comment 3: -** On this same page you refer to the indigent or core poor. It may be helpful to specifically define these terms. Furthermore, it could be of interest to touch about what percentage of the Ghanaian population is considered indigent or core poor.

**Authors’ response:** The term “indigent” has been defined in-text and a statistic for the proportion of Ghanaian populace considered as indigent/core poor has been given. The new text reads as:

*“…****the indigent or extreme poor, estimated to be about 8.2 percent of Ghana’s population*** *in 2016/17 [11] and those identified by the minster for employment and social welfare as needing support [12]. The indigent as defined by Act 650 (2003), which has been repealed by Act 852* *of 2012 refers to* ***“a person who does not have a visible or adequate means of income or who does not have a person to support him or her”.*** See **lines 47-51 on pages 2-3** of the tracked changes version of the manuscript

**Comment 4:** -On page 4, you use the term 'rare illness episodes'. I assume here you are referring to rare episodes of illness, rephrasing this may help avoid any confusion, as this could upon first glance be seen as referring to illnesses that are rare or uncommon, such as cancers. Though please note this is more of a style suggestion and this change is not essential as I think the point is well understand via the context.

**Authors’ response**: We have replaced “rare illness episodes” with “rare episodes of illness” as suggested. The sentence is now:

*“Other reasons included* ***rare episodes of illness****, limited benefits”* in **line 75, page 4** of the tracked changes version of the manuscript

**Comment 5:** - One page 12, I would use remain consistent and use the term "symptom persistence" both in the body of the first paragraph and the title of the next. I also feel "symptom persistence" sounds more natural in English.

**Authors’ response**: “Symptom persistence” has been substituted into the text as it reads and sounds better. The texts now read as:

*“These they referred to as illness severity,* ***symptom persistence*** *and affordability of health care cost.”*

*“Severity of illness and* ***symptom persistence*** *emerged as crucial”* **in lines 320 and 322 on page 14** of the tracked changes version of the manuscript.

**Comment 6:** - On page 24 under conclusions, the following sentence needs a few corrections: "The uninsured face a major challenge of financing their health care cost, while (not whiles) the insured do not...". Moreover, I would not use the pronoun "her" to refer to Ghana. "If this persists, Ghana may not be able to meet its..."

**Authors’ response**: “Whiles” has been replaced by “while” and the text now reads as:

*“The uninsured face a major challenge of financing their health care cost while the insured do not have all their healthcare expenditures covered”*

Additionally, in the subsequent sentence, we have replaced “her” with “the”:

*“If this persists, Ghana may not be able to meet* ***the*** *sustainable development goals of ensuring”*. See **lines 845 and 849 on page 31** of the tracked changes version of the manuscript.

**Comment 7:** -Under acknowledgements I would more directly state, "We thank all the people and hospital staff..."

**Authors’ response**: The statement of acknowledgments has been rewritten as *“We thank all the people and hospital staff who participated in the study.”* See **line 870 on page 32** of the tracked changes version of the manuscript.

**Comment 8:** -I feel some parts of the text were fairly repetitive. I might review to see where I could cut out repeated comments and replace the repeated points with sections where you could suggest or explore solutions to all the problems well-detailed in the article regarding the failure of the NHIS system to serve its intended purpose.

**Authors’ response**: We have reviewed and cut down on repeated text.

**Comment 9:** - I would be sure to mention setbacks as well. Discuss briefly sample size, biases, etc.

**Authors’ response**: We have stated study limitations on page 31 of the revised manuscript. We have briefly discussed sample size on pages 9 and 10 of the tracked changes version of the manuscript.

**REVIEWER # 4:**

**Comment 1:**

The introduction is quite lengthy and could be more focused.
**Authors’ response**: Superfluous information have been removed to focus the scope of the introduction.

**Comment 2:** In the 4th paragraph of the introduction the authors discuss the NHIS and the potential problems with this. In the subsequent paragraphs they explain what the NHIS is. The introduction would be greatly improved if they explained what the NHIS is before discussing the problems.

**Authors’ response**: Thank you very much for this useful and critical observation. We have now reorganize and restructure the introduction to explain what the NHIS is before discussing the problems as seen on **pages 1-6** of the tracked changes version of the manuscript.

**Comment 3:** Anderson healthcare utilisation model: This was discussed during the introduction but was not mentioned anywhere else in the paper and so it is unclear what relevance this has to this particular study? If the authors are keen to use this model in their study, they need to provide more information about how their data collection was developed using this model and whether or not the results align to this model.

**Authors’ response**: The results and discussion sections has been modified to reflect the Andersen model of health care utilization. In introducing the results section, we have reworded it as:

*“The findings of the study are presented below according to the predisposing, enabling and need factors as conceptualized by Andersen [30]. The predisposing factors consists of…”*. See **lines 298-301 on page 13** of the tracked changes version of the manuscript. Also, **see lines 707-710, 718-719 on page 26; 751-762; 802-809, and 827-835 on pages 27-31** of the tracked changes version of the manuscript.

**Comment 4:** Aims and objectives: It would be useful if the authors could include a clear statement about the aims and objectives at the end of the introduction. E.g. “the purpose of this study was to compare the experiences of NHIS insured and uninsured in accessing and utilizing healthcare services. Specifically, we aimed to….”

**Authors’ response**: The aims and objectives have been incorporated into the text as follows:

*The purpose of this study was to compare the experiences of NHIS insured and uninsured in accessing and utilizing healthcare services. Specifically, we aimed to explore factors motivating accessibility and utilization of health care services among NHIS subscribers and non-subscribers in the Hohoe Municipality; to assess barriers to accessibility and utilization of health care services by NHIS subscribers and non-subscribers. The study also sought to ascertain possible disparities in health care services provided to NHIS subscribers and non- subscribers; and to assess the views of service providers on the experiences of NHIS subscribers and non-subscribers in accessing and utilizing health care services****.* See lines 126-133 on page 6** of the tracked changes version of the manuscript.

**Comment 5:** The authors state that they chose five communities from within the Hohoe-sub municipality. Why were these five communities and are they representative (e.g. different socioeconomic communities) of the wider municipality?

**Authors’ response**: the reason for selecting those five communities has been given as follows:

*The selection of the five communities was also informed by the cosmopolitan nature of the municipality with different ethnic groups, socio-economic activities and demographic characteristics. This was, therefore, to give a cross-sectional representation of the wider Hohoe communities.* See **lines 217-221 on page 9** of the tracked changes version of the manuscript.

**Comment 6:** The authors should provide specific information about what topics were covered in the interview schedule.

**Authors’ response**: Specific information regarding the main topics of the interview has been given. The text reads as:

*The interview guide captured the socio-demographic characteristics of study participants, the motivating factors and or the extent to which the health insurance made a difference to subscribers’ access to and utilization of health care, the challenges uninsured have in accessing and utilizing healthcare, and health care providers’ experiences of providing care to both insured and uninsured under the NHIS. Other topics included reasons for not subscribing to the NHIS, non-renewal of health insurance, recommendations for improving the NHIS, barriers to health care utilization and access.* See **lines 243-250 on pages 9-10** of the tracked changes version of the manuscript

**Comment 7:** More detail is required about how the analysis was conducted / how the themes were decided upon. E.g. How many of the authors conducted the analysis? Were the themes checked and agreed on by multiple authors?

**Authors’ response**: The data analysis procedure has been updated and detailed in **lines 264-286 on pages 11-12** of the tracked changes version of the manuscript.

**Comment 8:** The themes aren’t clearly defined, with a lot of overlap between them.
The authors split the themes into factors motivating health care access and barriers to healthcare access. However, affordability of health care cost appears to be both a barrier and a facilitator and by splitting the discussion between the two themes the importance of this finding seems to get lost. Perhaps the authors may consider discussing barriers and facilitators within themes rather than having them as the main themes. E.g. create a theme “affordability of health care cost” and then discuss the barriers and facilitators of this as well as the differences / similarities between the insured and uninsured.

**Authors’ response**: The suggestion in this comment has been included in the revised manuscript. We have created the theme “*Affordability of health care cost*”. See **page 15 ff.**

**Comment 9:** In the methods the authors mention that health workers were interviewed but there is no further information about what they were interviewed about and there is no mention of this in the results?

**Authors’ response**: The data obtained from health workers have been included in the results as in seen in the sub-heading *“Service Providers Views and Experiences of providing Care to NHIS Subscribers and Non-Subscribers”*. See **pages 21-23** of the tracked changes version of the manuscript.

**Comment 10:** Many of the issues discussed under “reasons for non-subscription and recommendations…” were discussed under other headings so this doesn’t seem like a separate theme. As mentioned above, these issues can be discussed under more specific themes.

**Authors’ response**: This has now been made more specific **on pages 23-24** of the tracked changes version of the manuscript.

**Comment 11:** At the start of the discussion the authors mention views of health professionals but these weren’t actually discussed in the results.

**Authors’ response**: This has been rectified as is seen in the Results sub-heading “*Service Providers Views and Experiences of providing Care to NHIS Subscribers and Non-Subscribers”* on pages 21 23 of the tracked changes version of the manuscript. It has also been included in the discussion.

**Other revisions undertaken by authors’ during this revised submission**

The manuscript has been proofread for clarity and to also address any typographical and grammatical errors.
