# Supplementary material for: The shared experiences of insured members and the uninsured in health care access and utilization under Ghana’s national health insurance scheme: Evidence from the Hohoe Municipality
Source: PLoS One. 2020 Dec 23;15(12):e0244155. doi: 10.1371/journal.pone.0244155 (PMC7757881; doi:10.1371/journal.pone.0244155)
Supplement: Attachment — Submitted filename: Response to Reviewers.docx [file pone.0244155.s004.docx]

16^th^ November, 2020

The Academic Editor,

Plos One

Dear Academic Editor,

*Subject:* **Revised submission of manuscript for consideration**

We found both the Academic Editor and Reviewer(s) comments useful and very helpful in guiding us through the second round of minor revision. We are grateful for reviewing our paper and providing us with the opportunity to resubmit the revised manuscript.

In revising the manuscript, we took into account the Academic Editor and all reviewers’ suggestions of our revised submission. A point-by-point explanation of how we addressed the comments raised by the Academic Editor and reviewer(s) is detailed below.

All authors have read and approved this revised manuscript, and agreed with the publication of their names. There are no conflicts of interest.

We look forward to hearing from you on this matter.

Sincerely,

Adam Fusheini (PhD)

(Corresponding Author)

**Detail Revision notes on revised Manuscript**

## **Manuscript ID**: PONE-D-20-18400

**Title:** The shared experiences of insured members and the uninsured in health care access and utilization under Ghana's National Health Insurance Scheme: Evidence from the Hohoe Municipality

Below, we explain how we have addressed each of the comments of the reviewers.

- *All proper nouns including names of organizations, places, titles of people, names of journals in the reference list, etc need to be capitalized. These include Sustainable Development Goals (SDGs), Minister for Employment and Social Welfare, Hohoe sub-Municipality etc*

**Authors Response:** This has now been addressed throughout the manuscript in tracked changes. See the **Revised Manuscript with Track Changes.**

- *Please indicate the source of the figure used and the authorization obtained to use it in your manuscript.*

We did indicate the source of the manuscript in the figure title in the initial and first round revised versions on page 6 with reference [31]. However, due to authorization issues, we have decided to remove the figure in the revised manuscript.

- *The figure title normally goes below the figure, not above*

Thank you for drawing our attention but in both the initial original submission and first round revision, the title of the figure was below where the figure was to be inserted.

- *Anderson uses "enabling factors" in the model so please use same consistently throughout the paper.*

Thank you very much for the comment. We have now used “enabling factors” throughout the paper.

We hope we have addressed the comments in the manuscript to the satisfaction of both the Academic Editor and the reviewer(s).

Once again, thank you for giving us the opportunity to revise the manuscript.
