# Supplementary material for: The shared experiences of insured members and the uninsured in health care access and utilization under Ghana’s national health insurance scheme: Evidence from the Hohoe Municipality
Source: PLoS One. 2020 Dec 23;15(12):e0244155. doi: 10.1371/journal.pone.0244155 (PMC7757881; doi:10.1371/journal.pone.0244155)
Supplement: Attachment — Submitted filename: Response to Reviewers_Dec 2020.docx [file pone.0244155.s005.docx]

**Detail Revision notes on revised Manuscript**

## **Manuscript ID**: PONE-D-20-18400

**Title:** The shared experiences of insured members and the uninsured in health care access and utilization under Ghana's National Health Insurance Scheme: Evidence from the Hohoe Municipality

Below, we explain how we have addressed each of the points raised by the academic editor and reviewer(s).

**Authors Response:** The issues raised have now been addressed throughout the manuscript in tracked changes. See the **Revised Manuscript with Track Changes.**

- *Your manuscript needs a lot of editing to meet the quality standards of this journal. Hence please pay attention to the following and all related instances in your manuscript to improve its overall editorial quality suitable for publication in this journal:*

1) **Spellings--eg. Minister not Minster** *(This has now been corrected. See line 45, page 2 of the revised manuscript with tracked changes)*

2) **Volta Region is a proper noun so please write it as written here; kindly check all such instances of region and district names etc which should be written as proper nouns** *(This has now been corrected throughout the revised manuscript and all other proper nouns written in the correct manner)*

3) **Health Care Service Utilization Model is a proper noun so to be written as shown** *(This has now been written as shown in the revised manuscript. See lines 111-113 on page 5 of the revised manuscript wit tracked changes, and also on pages 22 and 24).*

4) **Hohoe sub-municipality is the right way of writing this; please correct all instances of Hohoe-sub municipal etc** *(All instances of Hohoe-sub municipal has been corrected throughout the manuscript. See the revised tracked changed version).*

5) **Please be consistent with numbers in your manuscript. Some parts of the manuscript have the numbers written in figures, other parts written out in words.** *(This has now been revised in the manuscript. See the revised tracked change version).*

6) **Low-And Middle-Income-Countries  should be written as low and middle-income countries (LMIC)** *(This has been corrected. Please, see line 4 on page 1 of the revised tracked change version).*

7) **Also please be consistent with the formatting of your headings and sub-headings throughout the manuscript, ensuring that sub-headings at the same level, eg all first-level sub-headings or all second-level sub-headings in the manuscript have the same formatting style, eg. capitalization, use of bolding etc** *(This has been changed in the revised version of the manuscript-see in tracked changes).*

8) **Journal  names in the reference list are usually in italics to make them stand out.** (All the journal names are now in Italics. See the reference list in the tracked change version).

We hope we have addressed the comments in the manuscript to the satisfaction of both the Academic Editor and the reviewer(s). The manuscripts has also been proofread.

Once again, thank you for giving us the opportunity to revise the manuscript.
